# Efficacy of Toxfin in Mitigating Mycotoxin Effects in Laying Hens

**DOI:** 10.3390/ani16162613

**Published:** 2026-08-20

**Authors:** Adiel Vieira de Lima, José de Arimatéia de Freitas Pinto, Carlos Henrique do Nascimento, Aline Beatriz Rodrigues, Paloma Eduarda Lopes de Souza, Humberto de Araújo Brito Filho, Raiane dos Santos Silva, Luayne Morais Correa, Ricardo Romão Guerra, Edijanio Galdino da Silva, Apolônio Gomes Ribeiro, Juliana Cristina Ramos Rezende Arrais, Matheus Carlos Romeiro Miranda, Julia Pinto Piccoli, Fernando Guilherme Perazzo Costa

**Affiliations:** 1 Department of Animal Science, Federal University of Paraíba, PB-079 Highway, Areia 58397-000, PB, Brazil; 2 Department of Veterinary Sciences, Federal University of Paraíba, PB-079 Highway, Areia 58397-000, PB, Brazil; 3 Kemin Animal Nutrition & Health Division, Latin America, Rua Krebsfer 736, Valinhos 13279-450, SP, Brazil

**Keywords:** mycotoxin adsorbent, bentonite, fumonisin B1, commercial laying hens, sepiolite, T-2 toxin

## Abstract

**Simple Summary:**

Mycotoxins are harmful substances produced by fungi that commonly contaminate animal feed and can negatively affect the health and productivity of laying hens. Among them, T-2 toxin and fumonisin are particularly damaging, leading to reduced egg production and poor egg quality. This study evaluated whether a feed additive called Toxfin™ Dry, made from natural mineral compounds, could reduce the harmful effects of these toxins. A total of 480 laying hens were fed diets with or without contamination by these toxins and with or without the additive over a period of 112 days. Birds exposed to mycotoxins showed lower egg production, poorer eggshell quality, and signs of stress and liver damage. However, hens that received the additive showed clear improvements, with performance and health indicators closer to those of birds not exposed to toxins. These results suggest that Toxfin™ Dry can help protect laying hens from the negative effects of mycotoxins by reducing their absorption in the digestive system. This is important for improving animal welfare, maintaining egg production, and reducing economic losses in the poultry industry.

**Abstract:**

This study evaluated the effects of supplementation with Toxfin™ Dry, a mineral adsorbent composed of bentonite and sepiolite, on productive performance, egg quality, intestinal morphometry, serum biochemical parameters, antioxidant status, and sphingolipid metabolism of laying hens challenged with T-2 toxin and fumonisin. A total of 480 Hy-Line W-36 laying hens at 25 weeks of age were distributed in a completely randomized design with six treatments and eight replicates of 10 birds each, over 112 days divided into four 28-day cycles. Treatments consisted of a control diet, either uncontaminated or contaminated with 2 mg/kg T-2 toxin and 60 mg/kg fumonisin B1, with or without 1 kg/ton Toxfin™ Dry. Mycotoxin challenge reduced egg production (*p* < 0.0001), egg weight (*p* < 0.0001), egg mass (*p* < 0.0001), feed intake (*p* < 0.0001), eggshell strength (*p* < 0.0001) and thickness (*p* < 0.0001), Haugh unit (*p* < 0.0001), and villus ratio (*p* < 0.0001), while increasing inflammatory and hepatic biomarkers, malondialdehyde levels, and the sphinganine ratio, with more pronounced effects under combined contamination. Supplementation with Toxfin™ Dry mitigated most adverse effects, with responses approaching those of the control group. The adsorbent reduces intestinal mycotoxin bioavailability and helps maintain performance and physiological homeostasis in laying hens.

## 1. Introduction

Among the various factors affecting poultry productivity, contamination of feed ingredients and diets with mycotoxins represents one of the most relevant challenges for the global poultry industry. These compounds are secondary metabolites produced by filamentous fungi and may be simultaneously present in feedstuffs, making the occurrence of multiple mycotoxins within the same diet frequent [1]. This condition is particularly concerning because interactions among different mycotoxins may result in additive or synergistic effects, intensifying physiological and productive impairments compared with individual exposure.

Among the mycotoxins of greatest importance in poultry production, T-2 toxin and fumonisins stand out. T-2 toxin belongs to the trichothecene group and exerts its toxicity primarily through inhibition of protein synthesis, triggering ribosomal stress, apoptosis, immunosuppression, and lesions in tissues characterized by high cell turnover, such as the intestinal mucosa [2]. In contrast, fumonisins exhibit a distinct mechanism of action characterized by inhibition of the enzyme ceramide synthase, leading to alterations in sphingolipid metabolism, disruption of cell membranes, increased intestinal permeability, and impaired cellular signaling [3]. Together, these alterations promote the development of systemic inflammation, oxidative stress, hepatic dysfunction, and reduced nutrient utilization efficiency, negatively affecting productive performance and egg quality [4].

The intestine is one of the main target organs of these mycotoxins. Alterations in intestinal mucosal architecture reduce the absorptive surface and impair epithelial renewal, limiting nutrient utilization. In parallel, increased intestinal permeability favors the translocation of pro-inflammatory molecules into circulation, intensifying the immune response and increasing the metabolic cost required to maintain homeostasis [5]. In addition, disruption of sphingolipid metabolism caused by fumonisin exposure has been recognized as a key molecular event underlying impaired cellular function, and the sphinganine to sphingosine ratio (Sa/So) is considered a sensitive biomarker of exposure and toxicity [6].

In response to the challenges posed by mycotoxins, the use of adsorbents in animal nutrition has been established as one of the main strategies to reduce their bioavailability in the gastrointestinal tract. These additives act by forming physicochemical complexes with mycotoxins in the intestinal lumen, limiting their absorption and, consequently, their systemic effects [7]. Among the minerals used for this purpose, bentonite stands out due to its high adsorption capacity resulting from its lamellar structure and large surface area, whereas sepiolite presents a highly porous fibrous structure, contributing to increased retention capacity for different compounds [8]. The combination of these minerals may represent a promising alternative to mitigate the effects of mycotoxin co-occurrence in poultry diets.

Despite advances achieved with the use of mineral adsorbents, most available studies focus on evaluating zootechnical performance or isolated intestinal health parameters. Information integrating productive responses, egg quality, intestinal histomorphological changes, oxidative stress biomarkers, sphingolipid metabolism, and systemic biochemical indicators in laying hens subjected to simultaneous fumonisin and T-2 toxin challenge remains limited. An integrated understanding of these mechanisms is essential to elucidate how reducing mycotoxin bioavailability may preserve physiological homeostasis and minimize productive losses.

Therefore, this study aimed to evaluate the effects of supplementation with Toxfin™ Dry, a mineral adsorbent based on bentonite and sepiolite, on growth performance, egg quality, intestinal morphometry, biochemical parameters, antioxidant status, and sphingolipid metabolism of commercial laying hens challenged with T-2 toxin and fumonisin. It was hypothesized that supplementation with the adsorbent would reduce intestinal absorption of mycotoxins, preserving intestinal mucosal integrity, metabolic and oxidative homeostasis, and consequently minimizing the deleterious effects of mycotoxins on productive performance and egg quality.

## 2. Materials and Methods

### 2.1. Animals, Housing and Experimental Design

All experimental procedures were conducted in accordance with the ethical principles for the use of animals in research and were approved by the Animal Care and Use Committee (CEUA) of the Federal University of Paraíba (UFPB), under protocol number 6876111225.

The experiment was conducted at the Poultry Teaching and Research Unit of the Center for Agricultural Sciences, Campus II, Federal University of Paraíba, located in Areia, Paraíba, Brazil. The experimental period lasted 112 days, divided into four consecutive 28-day cycles.

A total of 480 commercial laying hens (Hy-Line W-36) at 25 weeks of age were used in a completely randomized design consisting of six treatments, with eight replicates of 10 birds each, totaling 80 birds per treatment. The experimental unit consisted of a cage containing 10 birds. Birds were housed in conventional cages equipped with trough feeders and nipple drinkers and were provided feed and water ad libitum throughout the experimental period. Bird management followed the recommendations of the Hy-Line W-36 management guide, maintaining an appropriate lighting program and environmental conditions for the laying phase.

### 2.2. Experimental Diets and Treatments

Experimental diets were formulated based on corn and soybean meal to meet the nutritional requirements of light laying hens during the production phase, according to the recommendations of the Hy-Line W-36 Management Guide. The ingredient composition and calculated nutrient levels of the experimental diets are presented in Table 1.

The experimental treatments consisted of CON (control diet); CON + TXF (control diet supplemented with the mycotoxin adsorbent Toxfin™ Dry); CON + T2 (control diet contaminated with T-2 toxin); CON + T2 + TXF (control diet contaminated with T-2 toxin and supplemented with Toxfin™ Dry); CON + FUM + T2 (control diet contaminated with fumonisins and T-2 toxin); and CON + FUM + T2 + TXF (control diet contaminated with fumonisins and T-2 toxin and supplemented with Toxfin™ Dry).

Toxfin™ Dry (Kemin Industries, Valinhos, SP, Brazil) is a commercial mycotoxin adsorbent composed of sepiolite (440 g/kg) and bentonite (550 g/kg), with a broad adsorption spectrum, particularly for aflatoxins and fumonisins. It was included in the diets at 1 kg/ton of feed, according to the manufacturer’s recommendation.

The mycotoxins used in the experiment were produced by semi-solid fermentation of corn grains using isolates of Fusarium verticillioides, a fumonisin producer, and Fusarium sporotrichioides, a T-2 toxin producer. For the production of contaminated material, 100 g of ground corn were placed in 500 mL Erlenmeyer flasks, moistened with 40 mL of distilled water, and sterilized in an autoclave at 121 °C for 30 min. After cooling, the grains were inoculated with 2 mL of a suspension containing 1 × 10^8^ spores/mL of each fungus, previously cultured on potato dextrose agar for 10 days. Incubation was carried out for 21 days under static conditions at 25 °C. At the end of the fermentation period, the material was dried in an oven at 57 °C and ground to a particle size smaller than 0.85 mm.

The contaminated material contained 1950 mg/kg of fumonisin B1 and 129.2 mg/kg of T-2 toxin. Based on these concentrations, 3.100 kg of the fumonisin-containing material and 1.548 kg of the T-2 toxin-containing material were added to each 100 kg of feed to obtain final concentrations of 60 mg/kg fumonisin B1 and 2 mg/kg T-2 toxin in the experimental diets.

### 2.3. Productive Performance

Productive performance variables were evaluated at the end of each 28-day experimental cycle, considering each replicate (cage containing 10 birds) as the experimental unit (*n* = 8 per treatment). The following variables were determined: feed intake (g/bird/day), egg production (%), average egg weight (g), egg mass (g/bird/day), feed conversion per egg mass (kg/kg), and feed conversion per dozen eggs (kg/dozen).

Egg production was recorded daily. Feed intake was calculated as the difference between the amount of feed offered and the leftovers at the end of each experimental cycle, with values corrected according to observed mortality. Mortality was recorded daily, and the number of birds that died during each experimental cycle was considered when correcting feed intake values. Average egg weight was determined by weighing all eggs produced during the last three days of each 28-day experimental cycle. The mean egg weight was calculated for each experimental unit (cage), with eight experimental units per treatment (*n* = 8). Egg mass was calculated by multiplying the number of eggs produced by the average egg weight of each replicate within the period, whereas feed conversion per egg mass and per dozen eggs were obtained from the ratio between feed intake and egg mass produced or the number of dozens of eggs produced, respectively.

### 2.4. Egg Quality

Egg quality was evaluated during the last three days of each experimental period. The analyzed variables included yolk, albumen, and shell percentages, shell thickness (mm), specific gravity (g/cm^3^), yolk color, and Haugh unit. Specific gravity was determined according to Freitas et al. [9], based on the Archimedes principle, and obtained by dividing egg weight in air by the weight of water when the egg was submerged, with correction for temperature.

Yolk color was determined visually using the DSM/Roche color fan (1 to 15), by comparing yolk coloration with the scale standards. The Haugh unit (HU) was determined by breaking the eggs onto a flat glass surface and measuring the height of the thick albumen (mm) using a digital caliper. Egg weight (g) was measured using a digital scale (precision of four decimal places—0.0001 g). The albumen height and egg weight values were subsequently applied to the equation proposed by Haugh [10]: HU = 100 × log (H + 7.57 − 1.7 W^0.37^), where H is the height of the thick albumen (mm) and W is the egg weight (g).

For percentage determination, yolk weight was obtained separately using a digital scale with 0.0001 g precision, and albumen weight was calculated as the difference between egg weight and the weights of yolk and shell. Eggshells were identified, dried in a forced-air oven at 55 to 60 °C for 24 h, and weighed. The percentages of yolk, albumen, and shell were calculated as the ratio between the average weight of each component and the average egg weight. Shell thickness was measured using a digital micrometer (Mitutoyo 422-230-32, Kawasaki, Japan), with a range of 0 to 25 mm and precision of 0.001 mm.

### 2.5. Serum Biochemical Analyses

At the end of the experimental period, blood samples were collected from eight birds per treatment, corresponding to one bird per experimental unit. Before blood collection, birds were subjected to a 6-h fasting period. Approximately 4 mL of blood was collected by jugular vein puncture using sterile 13 × 0.4 mm needles and transferred into dry tubes containing a clot activator (BD Vacutainer® Dry, Becton, Dickinson and Company, Franklin Lakes, NJ, USA). Samples were allowed to stand at room temperature for 30 min to allow clot formation and were then centrifuged at 3500 rpm for 10 min in a bench centrifuge (SL-702/RAF30, Solab, Piracicaba, SP, Brazil) to obtain the serum. Serum samples were subsequently subjected to biochemical analyses according to the methodology described by Lima et al. [11].

Serum concentrations of glucose (GLU; Ref. 133), triglycerides (TG; Ref. 87), total cholesterol (CHOL; Ref. 1082), HDL-cholesterol (HDL-C; Ref. 13), uric acid (UA; Ref. 140), urea (U; Ref. 1013), creatinine (CRE; Ref. 1010), total bilirubin (TB; Ref. 31), aspartate aminotransferase (AST; Ref. 1009), alanine aminotransferase (ALT; Ref. 1008), gamma-glutamyl transferase (GGT; Ref. 1058), alkaline phosphatase (ALP; Ref. 1011), total proteins (TP; Ref. 1085), albumin (ALB; Ref. 1007), creatine kinase (CK; Ref. 117), calcium (Ca; Ref. 1084), phosphorus (P; Ref. 12–200), rheumatoid factor (RF; Ref. 332), and C-reactive protein (CRP; Ref. 3005) were determined using an automatic biochemical analyzer (Sinnowa Sx-260; Sinnowa Medical Science & Technology Co., Ltd., Nanjing, China) and commercial kits (Labtest Diagnóstica S.A., Lagoa Santa, MG, Brazil), following the manufacturer’s recommendations.

### 2.6. Oxidative Stress and Sphingolipid Metabolism

Biomarkers related to oxidative stress were determined in serum samples obtained at the end of the experimental period. Samples were stored at −80 °C until analysis. Serum concentrations of superoxide dismutase (SOD; Chicken SOD ELISA Kit, Cat. No. LT-4488KLE24), catalase (CAT; Chicken CAT ELISA Kit, Cat. No. LT-6688KLE24), glutathione peroxidase (GPx; Chicken GPX1 ELISA Kit, Cat. No. LT-6488KLE24), and malondialdehyde (MDA; Chicken MDA ELISA Kit, Cat. No. LT-5488KLE24) were determined using commercial enzyme-linked immunosorbent assay (ELISA) kits (Life Technologies [India] Pvt. Ltd., Delhi, India), according to the methodology described by Cengiz et al. [12] and the respective manufacturer’s recommendations. Plate readings were performed using an xMark™ Microplate Absorbance Spectrophotometer (Bio-Rad Laboratories, Hercules, CA, USA), and concentrations were calculated from the standard curves provided with the respective kits.

Sphingolipid metabolism was evaluated by determining serum concentrations of sphinganine (Sa) and sphingosine (So). Sphingolipids were extracted according to the methodology described by Rauber et al. [13]. For this, 0.5 mL of serum was mixed with 1.5 mL of 0.8% potassium chloride (KCl) solution, 0.05 mL of potassium hydroxide (KOH) solution, and 4.0 mL of ethyl acetate. The mixture remained under gentle agitation for 20 min and was subsequently centrifuged for 10 min at 1800 rpm. After centrifugation, the supernatant was collected, dried, and resuspended in 0.5 mL of methanol. The obtained extract was analyzed by liquid chromatography coupled with tandem mass spectrometry (LC-MS/MS), using an API 5000 system (Applied Biosystems, Concord, ON, Canada), as described by Rauber et al. [13]. The sphinganine/sphingosine ratio (Sa/So) was calculated as the ratio between the serum concentrations of these metabolites. The obtained extract was analyzed by liquid chromatography coupled with tandem mass spectrometry (LC-MS/MS), using an API 5000 system (Applied Biosystems, Concord, ON, Canada). The sphinganine/sphingosine ratio (Sa/So) was calculated as the ratio between the serum concentrations of sphinganine and sphingosine.

### 2.7. Intestinal Morphometry

At the end of the experimental period, eight birds per treatment were selected for intestinal sample collection. Fragments of the duodenum, jejunum, and ileum were collected and immediately fixed in buffered formalin for 24 h. All intestinal fragments were subsequently subjected to the same histological processing and paraffin embedding procedures. Transverse sections of 5 µm thickness were prepared from each intestinal segment and stained using hematoxylin–eosin (H&E) and periodic acid–Schiff (PAS) techniques.

Duodenal samples were collected 4 cm after the ventriculus, whereas jejunal fragments were obtained from the middle region of the segment and ileal fragments from the terminal portion of the intestine. All fragments were sectioned transversely, allowing adequate visualization of the intestinal villi and lumen.

Digital images of the duodenum, jejunum, and ileum sections were obtained using an Olympus BX-60 optical microscope coupled to an image capture system and analyzed using Motic Image Plus 2.0 software. For each intestinal fragment, 18 well-oriented structures were selected for morphometric measurements. Villus height was measured from the villus–crypt junction to the apex of the villus, whereas villus width was determined as the mean of three measurements taken at different points along the villus. Crypt depth was measured from the base of the crypt to the villus–crypt junction. The villus:crypt ratio was calculated by dividing villus height by crypt depth. Villus absorptive area was estimated as the product of villus height and villus width. All measurements were performed using the digital image analysis software.

### 2.8. Statistical Analysis

Data were analyzed using a completely randomized design (CRD). The initial evaluation of treatment effects was performed by analysis of variance (ANOVA), using the following statistical model:

Yij = μ + Ti + εij

where

Yij = observed response variable in replicate j of treatment i;
  μ = overall mean;
  Ti = fixed effect of treatment i;
  εij = random error associated with the observation, assumed to be normally distributed.

When a significant treatment effect was detected, treatment means were compared using Tukey’s test at a 5% probability level. Statistical analyses were performed using the SAS software, version 9.4 (SAS Institute Inc., Cary, NC, USA).

## 3. Results

### 3.1. Productive Performance

As shown in Table 2, there was a significant effect of treatments on all evaluated productive performance variables. For egg production, the highest values were observed in the control + Toxfin™ Dry and control treatments, with no difference between them, whereas the lowest values occurred in the control + fumonisin + T-2 and control + T-2 treatments. Treatments containing Toxfin™ Dry associated with mycotoxins showed intermediate values (*p* < 0.0001).

Feed intake was higher in the control and control + Toxfin™ Dry treatments, not differing between them, whereas the lowest intake was observed in the control + fumonisin + T-2 treatment. Treatments with the association of mycotoxins and adsorbent showed intermediate values, being higher than those with mycotoxins alone (*p* < 0.0001).

For egg weight, the highest values were observed in the control and control + Toxfin™ Dry treatments, followed by the control + T-2 + Toxfin™ Dry treatment. The lowest values were recorded in the control + fumonisin + T-2 and control + T-2 treatments, with intermediate values in the control + fumonisin + T-2 + Toxfin™ Dry treatment (*p* < 0.0001).

Egg mass followed a similar trend to egg production, with higher values in the control and control + Toxfin™ Dry treatments and lower values in the control + fumonisin + T-2 and control + T-2 treatments. Treatments including Toxfin™ Dry associated with mycotoxins showed intermediate values (*p* < 0.0001).

For feed conversion per egg mass, the lowest values were observed in the control, control + Toxfin™ Dry, and control + T-2 + Toxfin™ Dry treatments, which did not differ from each other, whereas the highest values were recorded in the control + T-2 and control + fumonisin + T-2 treatments. The control + fumonisin + T-2 + Toxfin™ Dry treatment showed an intermediate value (*p* < 0.0001).

Feed conversion per dozen eggs showed lower values in the control, control + Toxfin™ Dry, and control + T-2 + Toxfin™ Dry treatments, with no difference between them, whereas the highest value was observed in the control + fumonisin + T-2 treatment. The remaining treatments showed intermediate values (*p* < 0.0001).

### 3.2. Egg Quality

As shown in Table 3, there was a significant effect of treatments on shell strength (*p* < 0.0001), shell thickness (*p* < 0.0001), Haugh unit (*p* < 0.0001), specific gravity (*p* < 0.0001), and albumen pH (*p* < 0.0001). No significant differences were observed for yolk color (*p* = 0.9622), yolk percentage (*p* = 0.0614), shell percentage (*p* = 0.2240), albumen percentage (*p* = 0.1493), and yolk pH (*p* = 0.8418).

For shell strength, the highest values were observed in the control treatment, followed by the control + Toxfin™ Dry, control + T-2 + Toxfin™ Dry, and control + fumonisin + T-2 + Toxfin™ Dry treatments. The lowest values were recorded in treatments containing mycotoxins without adsorbent, especially control + fumonisin + T-2.

Shell thickness showed higher values in the control and control + Toxfin™ Dry treatments, with no difference between them, whereas the lowest values were observed in the control + fumonisin + T-2 and control + T-2 treatments. Treatments containing Toxfin™ Dry associated with mycotoxins showed intermediate values.

For Haugh unit, the highest values occurred in the control and control + Toxfin™ Dry treatments, whereas the lowest values were recorded in the control + fumonisin + T-2 treatment. The control + T-2, control + T-2 + Toxfin™ Dry, and control + fumonisin + T-2 + Toxfin™ Dry treatments showed intermediate values.

Specific gravity showed a similar pattern to that observed for Haugh unit and shell thickness, with higher values in the control and control + Toxfin™ Dry treatments and lower values in the control + fumonisin + T-2 and control + T-2 treatments. Treatments including Toxfin™ Dry associated with mycotoxins showed intermediate results.

For albumen pH, the highest values were observed in the control + T-2 and control + fumonisin + T-2 treatments, whereas the lowest values occurred in the control, control + Toxfin™ Dry, control + T-2 + Toxfin™ Dry, and control + fumonisin + T-2 + Toxfin™ Dry treatments, which did not differ from each other.

### 3.3. Intestinal Morphometric

**Figure 1:**
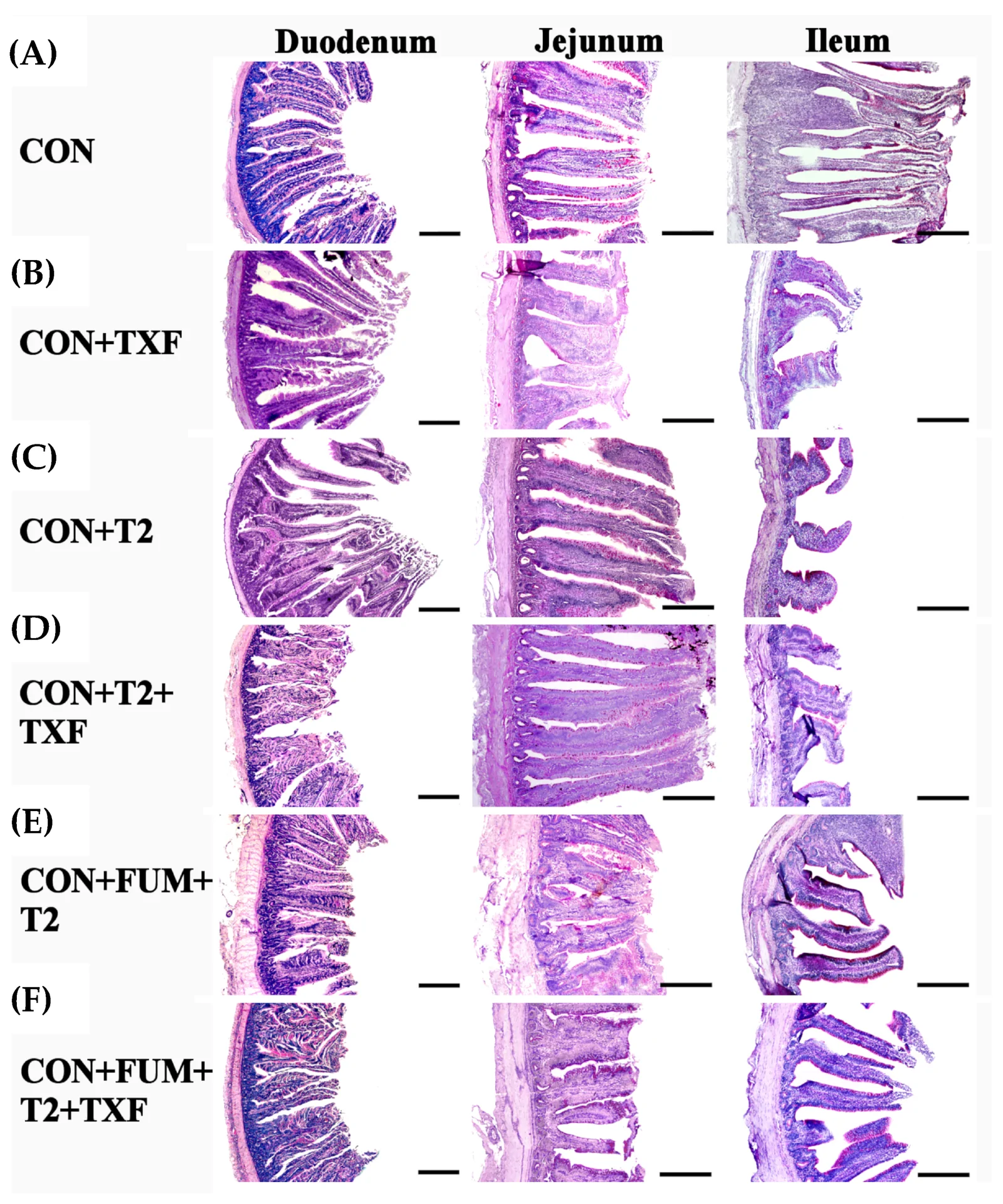
Photomicrographs of the duodenum, jejunum, and ileum of semi-heavy laying hens subjected to different dietary treatments. (**A**) CON; (**B**) CON + TXF; (**C**) CON + T2; (**D**) CON + T2 + TXF; (**E**) CON + FUM + T2; (**F**) CON + FUM + T2 + TXF. Note the intestinal morphology according to each intestinal segment and treatment. Staining: Periodic acid–Schiff (PAS) and hematoxylin. Scale bars: duodenum, 600 μm; jejunum and ileum, 300 μm.

As shown in Table 4 (Figure 1), there was a significant effect of treatments on intestinal morphometry variables evaluated in the duodenum, jejunum, and ileum.

In the duodenum, crypt depth showed higher values in the control + Toxfin™ Dry, control + T-2, and control + fumonisin + T-2 + Toxfin™ Dry treatments, whereas the lowest value was observed in the control treatment (*p* < 0.0001). For villus height, the highest value was recorded in the control + Toxfin™ Dry treatment, whereas the lowest value occurred in the control + fumonisin + T-2 treatment (*p* < 0.0001). Villus width showed a higher value in the control + T-2 + Toxfin™ Dry treatment and a lower value in the control + T-2 treatment (*p* < 0.0008). Villus area was higher in the control + Toxfin™ Dry treatment, differing from the other treatments (*p* < 0.0001). The Villus:crypt ratio showed higher values in the control + Toxfin™ Dry and control treatments, whereas the lowest value was recorded in the control + fumonisin + T-2 + Toxfin™ Dry treatment (*p* < 0.0001).

In the jejunum, crypt depth showed a higher value in the control + T-2 treatment and a lower value in the control treatment (*p* = 0.0141). For villus height, the highest value was observed in the control + T-2 + Toxfin™ Dry treatment, whereas the lowest value occurred in the control + Toxfin™ Dry treatment (*p* = 0.0002). Villus width showed higher values in the control and control + Toxfin™ Dry treatments, whereas the lowest values were recorded in the control + fumonisin + T-2 and control + fumonisin + T-2 + Toxfin™ Dry treatments (*p* < 0.0001). Villus area showed higher values in the control and control + T-2 + Toxfin™ Dry treatments, whereas the lowest values occurred in treatments containing fumonisin + T-2 (*p* < 0.0001). The Villus:crypt ratio showed a higher value in the control treatment and a lower value in the control + fumonisin + T-2 treatment (*p* = 0.0016).

In the ileum, crypt depth showed a higher value in the control + fumonisin + T-2 + Toxfin™ Dry treatment and a lower value in the control + Toxfin™ Dry treatment (*p* < 0.0001). For villus height, the highest values were observed in the control, control + T-2, control + T-2 + Toxfin™ Dry, and control + fumonisin + T-2 treatments, whereas the lowest values occurred in the control + Toxfin™ Dry and control + fumonisin + T-2 + Toxfin™ Dry treatments (*p* < 0.0001). Villus width showed a higher value in the control + fumonisin + T-2 + Toxfin™ Dry treatment and a lower value in the control + T-2 treatment (*p* = 0.0042). Villus area was higher in the control treatment, whereas the other treatments showed lower values and did not differ from each other (*p* < 0.0001). For the villous:crypt ratio, the treatments showed similar values, except for the control + fumonisin + T-2 + Toxfin™ Dry treatment, which showed the lowest value (*p* < 0.0001).

### 3.4. Biochemical, Oxidative Stress, and Sphingolipid Profile Parameters

As shown in Table 5, there was a significant effect of treatments on rheumatoid factor (*p* < 0.0001), C-reactive protein (*p* < 0.0001), HDL-cholesterol (*p* < 0.0001), glucose (*p* < 0.0001), urea (*p* = 0.0015), total bilirubin (*p* < 0.0001), aspartate aminotransferase (*p* = 0.0012), alanine aminotransferase (*p* = 0.0304), total proteins (*p* = 0.0001), albumin (*p* < 0.0001), creatine kinase (*p* < 0.0001), super-oxide dismutase (*p* < 0.0001), catalase (*p* < 0.0001), glutathione peroxidase (*p* < 0.0001), malondialdehyde (*p* < 0.0001), sphinganine (*p* < 0.0001), sphingosine (*p* < 0.0001), and the sphinganine ratio (*p* < 0.0001). No significant differences were observed for total cholesterol (*p* = 0.2539), triglycerides (*p* = 0.5474), uric acid (*p* = 0.2081), creatinine (*p* = 0.5363), gamma-glutamyl transferase (*p* = 0.1683), alkaline phosphatase (*p* = 0.9776), calcium (*p* = 0.719), and phosphorus (*p* = 0.2301).

Among the biochemical parameters, the highest values of rheumatoid factor and C-reactive protein were observed in the control + fumonisin + T-2 treatment, whereas the lowest values occurred in the control, control + Toxfin™ Dry, control + T-2 + Toxfin™ Dry, and control + fumonisin + T-2 + Toxfin™ Dry treatments. For HDL-cholesterol, the highest values were recorded in the control and control + Toxfin™ Dry treatments, whereas the lowest value occurred in the control + fumonisin + T-2 treatment.

Glucose showed the highest value in the control + fumonisin + T-2 treatment and the lowest values in the control and control + T-2 + Toxfin™ Dry treatments. Urea showed higher values in the control, control + Toxfin™ Dry, and control + fumonisin + T-2 + Toxfin™ Dry treatments, whereas the lowest value was observed in the control + T-2 treatment.

For total bilirubin and aspartate aminotransferase, the highest values were observed in the control + fumonisin + T-2 treatment, whereas the lowest values occurred in the control, control + Toxfin™ Dry, control + T-2 + Toxfin™ Dry, and control + fumonisin + T-2 + Toxfin™ Dry treatments. Alanine aminotransferase showed a lower value in the control + T-2 + Toxfin™ Dry treatment compared with the control treatment.

Total proteins and albumin showed higher values in the control + fumonisin + T-2 treatment, whereas the lowest values were observed in the control + T-2 + Toxfin™ Dry and control treatments, respectively. For creatine kinase, the highest value was recorded in the control + fumonisin + T-2 treatment, followed by treatments containing mycotoxins, whereas the lowest values occurred in the control and control + Toxfin™ Dry treatments.

In the antioxidant system, the highest values of superoxide dismutase, catalase, and glutathione peroxidase were observed in the control, control + Toxfin™ Dry, control + T-2 + Toxfin™ Dry, and control + fumonisin + T-2 + Toxfin™ Dry treatments, whereas the lowest values occurred in the control + fumonisin + T-2 treatment. In contrast, malondialdehyde showed the highest value in the control + fumonisin + T-2 treatment and the lowest values in the control and control + Toxfin™ Dry treatments.

For sphingolipid metabolism metabolites, the highest values of sphinganine and the sphinganine ratio were observed in the control + fumonisin + T-2 treatment, whereas the lowest values occurred in the control, control + Toxfin™ Dry, control + T-2 + Toxfin™ Dry, and control + fumonisin + T-2 + Toxfin™ Dry treatments. Sphingosine showed higher values in the control, control + Toxfin™ Dry, control + T-2, and control + T-2 + Toxfin™ Dry treatments, whereas the lowest values were observed in treatments containing fumonisin + T-2.

## 4. Discussions

The results obtained demonstrate that the mycotoxin challenge significantly impaired the productive performance of laying hens, as evidenced by reductions in egg production, feed intake, egg weight, and egg mass, in addition to a deterioration in feed conversion ratio. These effects were more pronounced when birds were simultaneously exposed to fumonisin and T-2 toxin, suggesting an additive or synergistic action between these mycotoxins. In contrast, supplementation with Toxfin™ Dry attenuated most of the observed impairments, bringing several variables closer to the values observed in the control group. The reduction in feed intake may represent one of the first events contributing to the decline in productive performance. T-2 toxin exerts cytotoxic effects on cells with high proliferative rates, particularly intestinal mucosal enterocytes, through inhibition of protein synthesis by binding to the 60S ribosomal subunit, triggering apoptosis, local inflammation, and loss of epithelial integrity [14,15]. In parallel, fumonisin directly interferes with sphingolipid metabolism through inhibition of ceramide synthase, altering cell membrane composition, impairing tight junction proteins, and increasing intestinal permeability [3,16,17]. These effects may reduce intestinal absorptive efficiency and nutrient utilization while increasing the energetic cost of maintaining cellular homeostasis. Consequently, reduced availability of energy and amino acids for egg formation may contribute to the reductions in egg production and egg mass observed in challenged birds [18].

This reasoning also explains the reduction observed in egg weight. The deposition of albumen proteins and yolk lipoproteins depends directly on the intestinal absorption of amino acids, lipids, and micronutrients, as well as hepatic biosynthetic capacity. The presence of mycotoxins likely reduced the efficiency of these processes through both impaired absorption and hepatotoxic effects resulting from intoxication. Thus, even in birds that maintained partial egg production, the lower availability of anabolic substrates limited the growth of egg components, resulting in lower final egg weight [19,20,21]. The deterioration in feed conversion ratio reinforces this physiological mechanism. Although part of this effect naturally results from reduced egg production, there is also an important metabolic component. Under mycotoxin intoxication conditions, energy demand increases due to tissue repair processes, acute-phase protein synthesis, immune system activation, and maintenance of oxidative balance. Consequently, a greater fraction of consumed metabolizable energy is diverted from production toward cellular homeostasis, reducing dietary utilization efficiency and explaining the higher feed conversion ratio observed in treatments containing mycotoxins, particularly when fumonisin and T-2 toxin were administered simultaneously [22].

The results obtained in the groups supplemented with Toxfin™ Dry suggest that the reduction in intestinal absorption of mycotoxins was sufficient to preserve, at least partially, gastrointestinal tract functionality. Bentonite and sepiolite, the main components of the adsorbent, exhibit high surface area and physicochemical adsorption capacity, forming relatively stable complexes with different mycotoxins throughout the digestive tract. Thus, a lower amount of these molecules reaches the systemic circulation, reducing their toxic effects on the intestine, liver, and other target tissues [23]. Importantly, the group supplemented only with Toxfin™ Dry showed performance similar to that of the control group for all evaluated variables, indicating that the adsorbent did not compromise dietary nutrient utilization or negatively affect bird productivity. This is relevant from a zootechnical perspective, since some mineral adsorbents may reduce the availability of vitamins, minerals, and other nutrients when they exhibit low binding selectivity [24].

Another relevant aspect is that the treatment containing only T-2 toxin reduced performance; however, these effects became significantly more pronounced when the toxin was associated with fumonisin. This response reinforces that the co-occurrence of mycotoxins represents a greater risk condition for commercial poultry. While T-2 toxin predominantly impairs epithelial renewal, protein synthesis, and immune response, fumonisin exerts marked effects on cell membrane integrity and lipid metabolism. The simultaneous action on distinct physiological pathways potentiates intestinal damage, intensifies cellular stress, and increases productive losses, explaining the greater magnitude of the effects observed in the present study [25].

Although productive performance represents an important zootechnical indicator, the observed results suggest that the recorded changes represent only the final manifestation of a more complex set of physiological events. The reduction in productive efficiency is likely associated with impairment of intestinal architecture, activation of inflammatory processes, increased oxidative stress, and metabolic alterations induced by mycotoxins. The changes observed in biochemical parameters corroborate these deleterious effects on systemic metabolism in laying hens and provide evidence that the reduction in productive performance resulted not only from impaired nutrient intake and absorption but also from important inflammatory and metabolic disturbances. Overall, the combination of fumonisin and T-2 toxin promoted the greatest alterations in serum biomarkers, whereas supplementation with Toxfin™ Dry was able to attenuate most of these responses, reinforcing its efficiency in reducing systemic exposure to mycotoxins.

The increased concentrations of C-reactive protein and rheumatoid factor in the challenged groups indicate activation of the systemic inflammatory response. C-reactive protein is an acute-phase protein produced mainly by the liver in response to the release of pro-inflammatory cytokines; therefore, its increase in birds subjected to mycotoxin intoxication can be interpreted as a reflection of innate immune system activation in response to cellular damage caused by these compounds. The concomitant increase in rheumatoid factor observed in this study reinforces this scenario of immune activation, suggesting that intoxication promoted alterations sufficiently intense to stimulate systemic inflammatory mechanisms. This response requires high amounts of energy and amino acids for the synthesis of acute-phase proteins and inflammatory mediators, diverting nutrients that, under physiological conditions, would be directed toward egg production.

The results also demonstrate an important impairment of hepatic function, mainly evidenced by the increase in aspartate aminotransferase (AST) and total bilirubin in the treatments containing fumonisin and T-2 toxin. AST is an intracellular enzyme present at high concentrations in hepatocytes, and its release into the circulation generally indicates increased membrane permeability or cellular injury. The increase in bilirubin may reflect reduced hepatic capacity for the metabolism and excretion of this pigment, indicating impaired liver function [26]. Considering that the liver is the main organ responsible for the synthesis of yolk lipoproteins, plasma proteins, and several metabolites involved in egg production, alterations in its functional integrity may directly contribute to the reduction in egg production, egg mass, and egg weight observed in this study.

Although alanine aminotransferase (ALT) presents lower diagnostic specificity in birds compared with mammals, its reduction in the treatment supplemented with Toxfin™ Dry in the presence of T-2 toxin may indicate a lower degree of hepatocellular injury or preservation of hepatocyte metabolic stability. However, considering the limited isolated applicability of this enzyme for hepatic evaluation in birds, its interpretation should be performed together with AST, bilirubin, and other biochemical indicators [27]. Another relevant result was the reduction in HDL-cholesterol concentrations in birds subjected to the simultaneous challenge with fumonisin and T-2 toxin. HDL plays a central role in reverse cholesterol transport and lipid redistribution to different tissues, including the ovary during yolk formation. The decrease in this lipoprotein may reflect alterations in hepatic lipoprotein synthesis or disturbances in lipid metabolism resulting from mycotoxin-induced hepatotoxicity [28]. In addition, fumonisin directly interferes with sphingolipid biosynthesis, which are important structural components of lipoprotein membranes, suggesting that alterations in this metabolism may additionally contribute to the modifications observed in the lipid profile.

The increase in serum glucose in birds subjected to the combined challenge also deserves attention. Under conditions of physiological stress, activation of the hypothalamic–pituitary–adrenal axis occurs, resulting in the release of corticosterone, the main glucocorticoid in birds. This hormone stimulates hepatic glycogenolysis and gluconeogenesis, increasing circulating glucose availability to meet the greater energy demand of tissues involved in the inflammatory response and cellular repair mechanisms [29]. Therefore, the hyperglycemia observed likely represents an adaptive response to the intense metabolic challenge imposed by mycotoxins, being consistent with the simultaneous reduction in productive performance.

The changes observed in total protein and albumin concentrations also suggest important modifications in protein metabolism. Although, in many situations of chronic hepatotoxicity, reductions in these parameters are expected due to decreased hepatic synthetic capacity, increases may occur in response to hemoconcentration, increased production of proteins related to the inflammatory process, or alterations in protein metabolism induced by intoxication [30]. Thus, these results should be interpreted together with the other hepatic and inflammatory markers, indicating that the association between fumonisin and T-2 toxin promoted important metabolic dysregulation.

The increase in creatine kinase (CK) observed mainly in birds exposed to mycotoxins reinforces the occurrence of systemic cellular injury. Although this enzyme is classically used as a marker of muscle damage, its elevation may be associated with increased cell membrane permeability resulting from oxidative stress and structural alterations induced by mycotoxins [30]. Considering that fumonisin directly compromises sphingolipid synthesis, which is essential for plasma membrane stability, it is plausible that part of the increase in CK reflects this mechanism of diffuse cellular injury.

Overall, the biochemical results demonstrate that the impairments observed in productive performance are a consequence of a complex pathophysiological process involving systemic inflammation, compromised hepatic function, alterations in energy metabolism, dysregulation of lipid metabolism, and increased cellular injury. The ability of Toxfin™ Dry to maintain most of these biomarkers close to the values observed in the control group reinforces that the reduction in intestinal absorption of mycotoxins was sufficient to preserve metabolic homeostasis in birds. This systemic protection constitutes one of the main mechanisms by which the adsorbent contributed to the maintenance of productivity, demonstrating that its benefits extend beyond simple luminal adsorption and directly reflect the preservation of target organ physiology.

The results related to the antioxidant system and sphingolipid metabolism provide important mechanistic evidence to explain the productive and metabolic alterations observed in birds subjected to mycotoxin challenge. The reduction in the activity of antioxidant enzymes, including superoxide dismutase (SOD), catalase (CAT), and glutathione peroxidase (GPx), associated with increased malondialdehyde (MDA) concentrations, demonstrates that simultaneous exposure to fumonisin and T-2 toxin promoted an intense redox imbalance, resulting in oxidative stress and lipid peroxidation. In contrast, supplementation with Toxfin™ Dry preserved antioxidant system activity and reduced MDA levels, indicating lower oxidative damage intensity and reinforcing its effectiveness in mitigating the toxic effects of mycotoxins.

Oxidative stress represents one of the main mechanisms involved in the toxicity of these mycotoxins. T-2 toxin promotes inhibition of protein synthesis, mitochondrial dysfunction, and increased production of reactive oxygen species (ROS), whereas fumonisin, by altering sphingolipid metabolism, compromises cell membrane stability and favors apoptosis and inflammatory processes [31]. When ROS generation exceeds endogenous antioxidant capacity, a state of redox imbalance is established, capable of affecting lipids, proteins, and nucleic acids, compromising cellular functionality and tissue integrity.

In this context, the reduction in SOD, CAT, and GPx activity observed in challenged birds indicates that endogenous antioxidant mechanisms were insufficient to neutralize the increased production of free radicals. These enzymes act in an integrated manner in cellular defense: SOD converts the superoxide radical into hydrogen peroxide, CAT decomposes this peroxide into water and oxygen, whereas GPx reduces lipid hydroperoxides and hydrogen peroxide using reduced glutathione as a cofactor. Therefore, the coordinated decrease in these enzymes demonstrates an overall impairment of antioxidant capacity, favoring the progression of oxidative damage [32].

As a consequence of this reduction in antioxidant defense, an increase in MDA concentrations was observed, representing one of the main end products of lipid peroxidation. MDA is widely used as a biomarker of oxidative damage to cell membranes, reflecting the degradation of polyunsaturated fatty acids present in the phospholipid bilayer. The increase in this metabolite indicates that the excess of free radicals promoted structural damage to cell membranes, altering their fluidity, permeability, and functionality [33]. This process has direct implications for bird physiology, as intact membranes are essential for intestinal nutrient absorption, maintenance of hepatic function, immune cell activity, and viability of reproductive tissues.

The preservation of antioxidant activity in the groups supplemented with Toxfin™ Dry likely results from the lower intestinal absorption of mycotoxins and, consequently, from the reduced systemic generation of reactive oxygen species. It is important to highlight that bentonite and sepiolite do not necessarily exert direct antioxidant activity. Their effect occurs mainly through an indirect mechanism by limiting mycotoxin bioavailability in the intestinal lumen, reducing the intensity of the inflammatory cascade and cellular processes responsible for free radical production [34].

The results regarding sphingolipid metabolism reinforce this interpretation and represent one of the most robust mechanistic pieces of evidence of the present study. Birds subjected to the challenge containing fumonisin showed increased concentrations of sphinganine (Sa), reduced sphingosine (So), and a consequent increase in the Sa/So ratio, alterations considered classical biomarkers of fumonisin intoxication [35]. These results confirm that fumonisin present in the diets effectively exerted its biological mechanism of action.

The explanation for this phenomenon lies in the high affinity of fumonisin for ceramide synthase, a key enzyme in the sphingolipid biosynthetic pathway. Inhibition of this enzyme prevents the incorporation of sphinganine into ceramide formation, causing its intracellular accumulation and, subsequently, increasing its circulating concentration. Simultaneously, there is a reduction in the availability of ceramides and sphingosine, molecules essential for maintaining cell membrane structure and signaling [36]. Considering that sphingolipids participate in the organization of membrane microdomains, cell adhesion, intracellular communication, and regulation of processes such as proliferation, differentiation, and apoptosis, alterations in this pathway profoundly compromise tissue homeostasis.

These modifications are particularly important for intestinal integrity. Sphingolipids are fundamental components of enterocyte membranes and proteins responsible for intercellular junctions. The reduction in ceramides favors increased intestinal permeability, allowing greater translocation of antigens and endotoxins into the circulation, which enhances activation of the systemic inflammatory response observed through increases in C-reactive protein and rheumatoid factor. In parallel, impairment of the intestinal barrier reduces digestive and nutrient absorption efficiency, establishing a direct link between sphingolipid metabolism, inflammation, and the reduction in productive performance observed in this study [37].

In addition, several metabolites of the sphingolipid pathway participate in the regulation of mitochondrial function and apoptosis. Sphinganine accumulation may favor mitochondrial dysfunction, increased production of reactive oxygen species, and activation of pro-apoptotic pathways, establishing a feedback loop between alterations in lipid metabolism and oxidative stress [38]. Therefore, the results of Sa/So and antioxidant biomarkers should not be interpreted separately, but rather as components of the same pathophysiological process triggered by fumonisin.

The reduction in sphinganine concentration and the Sa/So ratio in the groups supplemented with Toxfin™ Dry demonstrates that the adsorbent was able to limit intestinal absorption of fumonisin, reducing its interaction with ceramide synthase and partially preserving sphingolipid metabolism. This result has high biological relevance because it confirms that the benefits observed on performance, serum biochemistry, and antioxidant status did not result only from nonspecific responses, but from the attenuation of the main molecular mechanism of fumonisin toxicity. Thus, the set of results indicates that the protection provided by Toxfin™ Dry begins in the gastrointestinal tract, reducing mycotoxin bioavailability and interrupting a cascade of events involving sphingolipid metabolism dysfunction, oxidative stress, systemic inflammation, hepatic impairment, and, ultimately, losses in productive efficiency of laying hens.

The alterations observed in intestinal morphometry demonstrate that exposure to mycotoxins promoted remodeling of the intestinal mucosa, with distinct responses among the duodenum, jejunum, and ileum. Although some variables showed apparently heterogeneous behavior among segments, the overall results indicate impairment of intestinal architecture, particularly in birds subjected to the simultaneous challenge with fumonisin and T-2 toxin. These alterations have high physiological relevance because intestinal mucosal integrity represents one of the main determinants of digestive and absorptive efficiency in laying hens.

The intestinal epithelium is characterized by a high rate of cellular renewal and is particularly susceptible to the action of cytotoxic mycotoxins. T-2 toxin acts by inhibiting protein synthesis and disrupting the proliferation of cells located in intestinal crypts, whereas fumonisin compromises cell membrane integrity by altering sphingolipid metabolism, favoring apoptosis, disruption of intercellular junctions, and increased epithelial permeability [39]. Consequently, an imbalance between cellular loss and renewal occurs, resulting in modifications in villus and crypt architecture.

Villus height represents one of the main indicators of intestinal absorptive capacity, as more developed villi provide a greater contact surface between enterocytes and luminal contents. In the present study, the reduction in villus height and area in different intestinal segments, particularly in treatments containing fumonisin and T-2 toxin, suggests a decrease in the surface available for nutrient absorption. This reduction may partially explain the lower feed intake, reduced egg production, and impaired feed conversion ratio previously observed, since limited absorption compromises the supply of energy, amino acids, minerals, and vitamins required to sustain the high metabolic demand of egg production [40].

The alterations observed in crypt depth require careful interpretation. An increase in this parameter does not always represent an improvement in intestinal function; it is frequently associated with increased proliferative activity of stem cells as a compensatory mechanism in response to greater enterocyte loss at the villus tip. Under conditions of continuous injury, the intestinal mucosa increases the rate of cellular renewal in an attempt to repair the damaged epithelium, increasing the energy expenditure required for intestinal tissue maintenance [41]. Therefore, deeper crypts observed in some treatments may reflect an adaptive process in response to mycotoxin-induced damage rather than greater functional efficiency.

In this context, the villous–crypt ratio represents a more robust indicator of intestinal functionality because it simultaneously integrates absorption and cellular renewal processes. Higher ratios generally reflect an intact and efficient mucosa, whereas reductions in this variable indicate an imbalance between epithelial loss and regeneration. The decrease in the villous–crypt ratio observed mainly in treatments containing fumonisin demonstrates reduced functional efficiency of the intestinal mucosa, reinforcing the hypothesis of impaired absorptive capacity. This result is consistent with the alterations observed in zootechnical performance and strengthens the interpretation that the intestine represents one of the main target organs for the action of these mycotoxins.

Although Toxfin™ Dry did not completely restore all morphometric parameters, its supplementation promoted partial improvement in several variables, indicating preservation of intestinal architecture. This response likely results from the lower exposure of the mucosa to mycotoxins due to their adsorption in the intestinal lumen. Reduced contact between toxic compounds and the epithelium favors the maintenance of enterocyte integrity, decreases the intensity of the local inflammatory response, and preserves digestive and absorptive mechanisms. These findings are consistent with the results observed for performance, serum biochemistry, and oxidative stress, suggesting that intestinal protection represents one of the main mechanisms responsible for the beneficial effects of the adsorbent [42].

The alterations in egg quality also reflect the systemic effects promoted by mycotoxins. The reduction in shell resistance, shell thickness, and specific gravity demonstrates that intoxication impaired mineralization processes during egg formation. Calcium carbonate deposition in the shell gland depends on the adequate supply of calcium, phosphorus, bicarbonate, and metabolic energy, in addition to the coordinated activity of transport proteins and enzymes involved in calcification [43]. Thus, alterations in intestinal absorption and hepatic metabolism may reduce the availability of these components, resulting in structurally weaker eggshells. In addition to reduced mineral absorption, impaired eggshell quality may be related to the increased oxidative stress observed in intoxicated birds. Reactive oxygen species may alter uterine (shell gland) cell function, compromising the secretion of the organic matrix on which mineral deposition occurs. In parallel, systemic inflammatory status increases the demand for nutrients allocated to defense mechanisms, reducing their availability for reproductive processes [44].

The reduction in Haugh unit observed in birds subjected to the mycotoxin challenge indicates loss of internal egg quality, mainly due to alterations in the physicochemical properties of albumen. Haugh unit is directly related to albumen viscosity and protein integrity, especially ovomucin, whose synthesis depends on adequate protein metabolism and oviduct functionality [45,46]. Considering that mycotoxins promoted hepatic, inflammatory, and oxidative alterations, it is likely that these factors impaired the synthesis or stability of albumen proteins, explaining the reduction in this parameter.

The increase in albumen pH observed in birds exposed to mycotoxins also reinforces the deterioration of internal egg quality. Higher pH values are generally associated with the loss of albumen gel structure and reduced protein stability, favoring decreased viscosity and, consequently, lower Haugh unit values [47]. The fact that Toxfin™ Dry maintained these parameters close to those observed in the control group suggests that the preservation of metabolic and intestinal integrity contributed to maintaining internal egg quality.

On the other hand, the absence of differences in yolk, albumen, and shell percentages, as well as yolk color and yolk pH, indicates that mycotoxins exerted a greater influence on the structural quality of egg components than on their proportional distribution. These results suggest that, at the concentrations used, the challenge was sufficient to impair physiological processes related to eggshell formation and strength, as well as albumen protein quality, without significantly altering the relative composition of the main egg constituents.

Considering the results obtained as a whole, it becomes evident that intestinal integrity represents the central point of the physiological response observed in this study. Reduced nutrient absorption resulting from morphological alterations of the mucosa, combined with activation of systemic inflammation, increased oxidative stress, and alterations in sphingolipid metabolism, culminated in impaired hepatic function, reduced metabolic efficiency, and deterioration of productive performance and egg quality. By reducing intestinal mycotoxin bioavailability, Toxfin™ Dry interrupted this pathophysiological cascade at its initial stage, partially preserving intestinal and systemic homeostasis and, consequently, minimizing the negative effects of the fumonisin and T-2 toxin challenge.

## 5. Conclusions

The challenge with T-2 toxin, either alone or associated with fumonisin, impaired productive performance, egg quality, intestinal morphometry, and biochemical and oxidative balance in laying hens, with the most pronounced detrimental effects occurring during the co-occurrence of both mycotoxins. The inclusion of Toxfin™ Dry at a dosage of 1 kg/ton alleviated these effects, preserving egg production and egg mass, eggshell strength and thickness, intestinal mucosal integrity, antioxidant enzyme activity, and the sphinganine ratio, without impairing the performance of non-challenged birds. These results indicate that reducing intestinal mycotoxin bioavailability represents an effective strategy to mitigate the effects of fumonisin and T-2 toxin contamination in diets of commercial laying hens.

## Figures and Tables

**Table 1:** Ingredients and nutrient composition of experimental diets.

Ingredients	CON	CON + TXF	CON + T2	CON + T2 + TXF	CON + FUM + T2	CON + FUM + T2 + TXF
Corn	537.868	537.868	522.388	522.388	491.388	491.388
T-2 toxin-contaminated corn ^1^	0.000	0.000	15.480	15.480	15.480	15.480
Fumonisin-contaminated corn ^2^	0.000	0.000	0.000	0.000	31.000	31.000
Soybean meal	288.963	288.963	288.963	288.963	288.963	288.963
Soybean oil	48.312	48.312	48.312	48.312	48.312	48.312
Limestone	95.858	95.858	95.858	95.858	95.858	95.858
Dicalcium phosphate	17.678	17.678	17.678	17.678	17.678	17.678
Salt	2.964	2.964	2.964	2.964	2.964	2.964
Sodium bicarbonate	1.896	1.896	1.896	1.896	1.896	1.896
L-Lysine	0.044	0.044	0.044	0.044	0.044	0.044
DL-Methionine	2.417	2.417	2.417	2.417	2.417	2.417
Inert material ^3^	1.000	0.000	1.000	0.000	1.000	0.000
Toxfin™ Dry ^4^	0.000	1.000	0.000	1.000	0.000	1.000
Premix ^5^	3.000	3.000	3.000	3.000	3.000	3.000
Total	1000	1000	1000	1000	1000	1000
**Nutrient composition**						
Crude protein, %	17.50	17.50	17.50	17.50	17.50	17.50
Metabolizable energy, kcal/kg	2950	2950	2950	2950	2950	2950
Crude fiber, %	2.852	2.852	2.852	2.852	2.852	2.852
Neutral detergent fiber, %	11.352	11.352	11.352	11.352	11.352	11.352
Ash, %	2.482	2.482	2.482	2.482	2.482	2.482
Starch, %	36.010	36.010	36.010	36.010	36.010	36.010
Ether extract, %	7.046	7.046	7.046	7.046	7.046	7.046
Calcium, %	4.150	4.150	4.150	4.150	4.150	4.150
Available phosphorus, %	0.420	0.420	0.420	0.420	0.420	0.420
Sodium, %	0.180	0.180	0.180	0.180	0.180	0.180
Chloride, %	0.239	0.239	0.239	0.239	0.239	0.239
Potassium, %	0.701	0.701	0.701	0.701	0.701	0.701
Dietary electrolyte balance, mEq/kg	190.000	190.000	190.000	190.000	190.000	190.000
Digestible lysine, %	0.810	0.810	0.810	0.810	0.810	0.810
Digestible methionine, %	0.491	0.491	0.491	0.491	0.491	0.491
Digestible methionine + cysteine, %	0.730	0.730	0.730	0.730	0.730	0.730
Digestible threonine, %	0.591	0.591	0.591	0.591	0.591	0.591
Digestible tryptophan, %	0.194	0.194	0.194	0.194	0.194	0.194
Digestible valine, %	0.743	0.743	0.743	0.743	0.743	0.743
Digestible arginine, %	1.052	1.052	1.052	1.052	1.052	1.052
Digestible isoleucine, %	0.859	0.859	0.859	0.859	0.859	0.859

CON = control; TXF = Toxfin™ Dry; T2 = T-2 toxin; FUM = fumonisin. ^1^ T-2 toxin-contaminated corn, obtained by semi-solid fermentation with *Fusarium sporotrichioides*, containing 129.2 mg of T-2 toxin/kg. It was included in the diets at a ratio of 15.48 g/kg (1.548 kg/t), resulting in a final concentration of 2 mg of T-2 toxin/kg of feed. ^2^ Fumonisin B_1_-contaminated corn, obtained by semi-solid fermentation with *Fusarium verticillioides*, containing 1950 mg of fumonisin B_1_/kg. It was included in the diets at a ratio of 31.00 g/kg (3.100 kg/t), resulting in a final concentration of 60 mg of fumonisin B_1_/kg of feed. ^3^ Kaolin. ^4^ Toxfin™ Dry (Kemin Industries, Valinhos, SP, Brazil), a mycotoxin adsorbent composed of 440 g/kg sepiolite and 550 g/kg bentonite, included in the diets at 1 kg/ton of feed, according to the manufacturer’s recommendation. ^5^ Vitamin–mineral premix. Provided per kilogram of feed: 66 mg of Fe (FeSO_4_·7H_2_O), 83 mg of Zn (ZnSO_4_·7H_2_O), 80 mg of Mn (MnSO_4_·H_2_O), 1 mg of I (KI), and 6.8 mg of Cu (CuSO_4_·5H_2_O); 11,700 IU of vitamin A, 3600 IU of vitamin D_3_, 21 IU of vitamin E, 4.2 mg of vitamin K_3_, 3.0 mg of vitamin B_1_, 10.2 mg of vitamin B_2_, 0.9 mg of folic acid, 15 mg of pantothenic acid, 45 mg of niacin, 5.4 mg of vitamin B_6_, 24 μg of vitamin B_12_, and 150 μg of biotin.

**Table 2:** Productive performance of laying hens fed diets containing mycotoxins and the mycotoxin adsorbent (Toxfin™ Dry).

Variables	CON	CON + TXF	CON + T2	CON + T2 + TXF	CON + FUM + T2	CON + FUM + T2 + TXF	CV (%)	*p*-Value
EP	92.22 ^a,b^	92.80 ^a^	78.53 ^d^	89.35 ^b^	74.06 ^e^	85.32 ^c^	2.27	<0.0001
FI	113.32 ^a^	112.47 ^a^	103.10 ^c^	108.61 ^b^	97.65 ^d^	107.44 ^b^	1.29	<0.0001
EW	61.96 ^a^	61.97 ^a^	58.05 ^d^	61.00 ^b^	56.16 ^e^	59.12 ^c^	0.88	<0.0001
EM	57.14 ^a^	57.52 ^a^	45.59 ^d^	54.50 ^b^	41.60 ^e^	50.44 ^c^	2.47	<0.0001
FCRm	1.98 ^c^	1.95 ^c^	2.26 ^a^	1.99 ^c^	2.35 ^a^	2.13 ^b^	3.00	<0.0001
FCRdz	1.47 ^c^	1.45 ^c^	1.57 ^a,b^	1.46 ^c^	1.58 ^a^	1.51 ^b,c^	2.78	<0.0001

CON = control; TXF = Toxfin™ Dry; T2 = T-2 toxin; FUM = fumonisin; EP = egg production (%); FI = feed intake (g/bird/day); EW = egg weight (g); EM = egg mass (g/bird/day); FCRm = feed conversion ratio based on egg mass (g/g); FCRdz = feed conversion ratio per dozen eggs (kg/dozen). Means within a row followed by different letters differ according to Tukey’s test (*p* < 0.05).

**Table 3:** Egg quality of laying hens fed diets contaminated with mycotoxins and supplemented with a mycotoxin adsorbent (Toxfin™ Dry).

Variables	CON	CON + TXF	CON + T-2	CON + T-2 + TXF	CON + FUM + T-2	CON + FUM + T-2 + TXF	CV (%)	*p*-Value
SR	4.89 ^a^	4.76 ^b^	3.86 ^e^	4.42 ^c^	3.51 ^f^	4.09 ^d^	5.97	<0.0001
YC	4.03	4.01	4.02	4.00	4.02	4.00	9.91	0.9622
ST	0.334 ^a^	0.334 ^a^	0.306 ^d^	0.322 ^b^	0.295 ^e^	0.316 ^c^	3.18	<0.0001
HU	95.75 ^a^	95.04 ^a^	90.02 ^d^	92.86 ^b^	86.98 ^e^	91.10 ^c^	3.83	<0.0001
SG	1.0889 ^a^	1.0881 ^a^	1.0741 ^d^	1.0816 ^b^	1.0661 ^e^	1.0781 ^c^	0.37	<0.0001
YP	27.52	27.42	27.46	27.39	27.60	27.65	4.29	0.0614
SP	10.27	10.29	10.33	10.26	10.28	10.25	3.89	0.2240
AP	62.21	62.28	62.20	62.35	62.10	62.11	2.07	0.1493
Yolk pH	6.10	6.10	6.10	6.09	6.10	6.10	1.30	0.8418
Albumen pH	7.80 ^b^	7.79 ^b^	8.01 ^a^	7.80 ^b^	7.99 ^a^	7.82 ^b^	1.86	<0.0001

CON = control; TXF = Toxfin™ Dry; T-2 = T-2 toxin; FUM = fumonisin; SR = shell resistance (kg/f); YC = yolk color; ST = shell thickness (mm); HU = Haugh unit; SG = specific gravity (g/cm^3^); YP = yolk percentage (%); SP = shell percentage (%); AP = albumen percentage (%). Means within a row followed by different letters differ according to Tukey’s test (*p* < 0.05).

**Table 4:** Intestinal morphometric characteristics of laying hens fed diets containing mycotoxins and a mycotoxin adsorbent (Toxfin™ Dry).

Treatment	CON	CON + TXF	CON + T-2	CON + T-2 + TXF	CON + FUM + T-2	CON + FUM + T-2 + TXF	CV (%)	*p*-Value
**Duodenum**								
Crypt depth (μm)	70.37 ^b^	78.48 ^a^	81.45 ^a^	73.48 ^a,b^	74.31 ^a,b^	81.56 ^a^	<0.0001	25.58
Villus height (μm)	1513.67 ^b,c^	1802.11 ^a^	1608.54 ^b^	1512.28 ^b,c^	1436.28 ^c^	1484.93 ^b,c^	<0.0001	20.22
Villus width (μm)	202.81 ^ab^	192.62 ^a,b,c^	182.27 ^c^	211.24 ^a^	189.16 ^b,c^	193.15 ^a,b,c^	0.0008	25.06
Villus area (μm^2^)	310.729 ^b^	362.898 ^a^	293.825 ^b^	318.263 ^a,b^	279.609 ^b^	289.270 ^b^	<0.0001	36.35
Villus:crypt ratio (μm)	22.87 ^a,b^	24.26 ^a^	21.10 ^b,c^	21.89 ^a,b^	20.66 ^b,c^	18.96 ^c^	<0.0001	31.53
**Jejunum**								
Crypt depth (μm)	61.89 ^b^	62.87 ^a,b^	70.29 ^a^	66.25 ^a,b^	68.83 ^a,b^	68.73 ^a,b^	0.0141	29.89
Villus height (μm)	783.15 ^a,b,c^	703.61 ^c^	798.58 ^a,b^	836.80 ^a^	716.18 ^b,c^	742.05 ^b,c^	0.0002	29.40
Villus width (μm)	143.04 ^a^	135.12 ^a,b^	127.28 ^a,b^	134.42 ^a,b^	118.77 ^b^	119.13 ^b^	<0.0001	31.86
Villus area (μm^2^)	112.175 ^a^	98.456 ^a,b^	104.082 ^a,b^	114.324 ^a^	84.647 ^b^	89.763 ^b^	<0.0001	48.84
Villus:crypt ratio (μm)	13.49 ^a^	12.13 ^a,b,c^	12.41 ^a,b,c^	13.27 ^a,b^	11.23 ^c^	11.39 ^b,c^	0.0016	37.33
**Ileum**								
Crypt depth (μm)	63.63 ^b^	50.86 ^c^	61.42 ^b^	63.62 ^b^	66.31 ^a,b^	73.44 ^a^	<0.0001	28.34
Villus height (μm)	705.04 ^a^	536.01 ^b^	659.88 ^a^	694.85 ^a^	661.83 ^a^	586.27 ^b^	<0.0001	26.80
Villus width (μm)	126.07 ^a,b^	120.06 ^a,b^	109.57 ^b^	111.96 ^a,b^	122.10 ^a,b^	129.49 ^a^	0.0042	34.22
Villus area (μm^2^)	116.319 ^a^	64.746 ^b^	70.015 ^b^	77.596 ^b^	79.014 ^b^	78.153 ^b^	<0.0001	51.96
Villus:crypt ratio (μm)	11.56 ^a^	11.58 ^a^	11.34 ^a^	11.73 ^a^	10.64 ^a^	8.37 ^b^	<0.0001	37.25

CON = control; TXF = Toxfin™ Dry; T-2 = T-2 toxin; FUM = fumonisin; Different letters within rows indicate significant differences according to Tukey’s test (*p* < 0.05).

**Table 5:** Biochemical, oxidative stress, and sphingolipid profile parameters of laying hens fed diets containing mycotoxins and a mycotoxin adsorbent (Toxfin™).

Variables	CON	CON + TXF	CON + T2	CON + T2 + TXF	CON + FUM + T2	CON + FUM + T2 + TXF	*p*-Value	CV (%)
Biochemical parameters								
**RF**	5.78 ^c^	5.76 ^c^	7.24 ^b^	5.92 ^c^	9.86 ^a^	6.08 ^c^	<0.0001	4.29
**CRP**	210.71 ^c,d^	208.94 ^d^	268.34 ^b^	219.87 ^c,d^	345.93 ^a^	228.14 ^c^	<0.0001	4.91
**HDL-C**	43.89 ^a^	44.05 ^a^	39.65 ^b^	43.12 ^a^	34.18 ^c^	42.36 ^a,b^	<0.0001	5.34
**Chol**	89	106.25	85.62	85.5	144.75	130.38	0.2539	57.25
**TG**	789.25	711.88	944.88	829.63	1109.88	960.63	0.5474	50.26
**Glu**	196.00 ^c^	236.25 ^a,b^	214.00 ^b,c^	192.50 ^c^	247.25 ^a^	221.25 ^a,b,c^	<0.0001	10.05
**UA**	3.31	2.89	2.46	2.6	4.9	6.64	0.2081	99.94
**U**	5.50 ^a^	5.75 ^a^	2.62 ^b^	5.00 ^a,b^	4.37 ^a,b^	6.62 ^a^	0.0015	35.75
**CRE**	0.53	0.54	0.53	0.54	0.58	0.56	0.5363	11.8
**TB**	1.18 ^c^	1.17 ^c^	1.46 ^b^	1.21 ^c^	1.88 ^a^	1.25 ^c^	<0.0001	4.92
**AST**	177.13 ^b^	200.13 ^a,b^	177.00 ^b^	164.63 ^b^	234.63 ^a^	172.13 ^b^	0.0012	17.59
**ALT**	5.12 ^a^	3.87 ^a,b^	3.50 ^a,b^	3.00 ^b^	4.62 ^a,b^	4.50 ^a,b^	0.0304	32.69
**GGT**	25.25	20.87	19.5	26.5	18.25	38.75	0.1683	66.8
**ALP**	258.5	252.75	258.88	243.63	263.63	269.38	0.9776	24.87
**TP**	3.20 ^b,c^	3.69 ^a,b^	3.54 ^a,b,c^	2.79 ^c^	4.26 ^a^	3.96 ^a,b^	0.0001	16.25
**Alb**	1.70 ^c,d^	1.98 ^a,b,c^	1.92 ^b,c^	1.49 ^d^	2.30 ^a^	2.15 ^a,b^	<0.0001	12.44
**CK**	202.50 ^e^	191.63 ^e^	482.50 ^b^	336.88 ^d^	579.38 ^a^	390.00 ^c^	<0.0001	5.98
**Ca**	24.21	20.73	26.65	21.56	24.46	26.72	0.719	34.16
**P**	2.94	2.64	2.75	2.65	3.55	3.8	0.2301	38.35
Oxidative stress and antioxidant system								
**SOD**	127.00 ^a^	128.42 ^a^	112.76 ^b^	124.96 ^a^	95.42 ^c^	121.84 ^a^	<0.0001	3.93
**CAT**	29.60 ^a^	29.92 ^a^	25.87 ^b^	28.94 ^a^	21.34 ^c^	28.71 ^a^	<0.0001	4.34
**GPx**	216.00 ^a^	218.12 ^a^	188.73 ^b^	210.62 ^a^	162.91 ^c^	206.84 ^a^	<0.0001	5.5
**MDA**	5.97 ^c,d^	5.81 ^d^	7.48 ^b^	6.12 ^c,d^	9.82 ^a^	6.38 ^c^	<0.0001	5.19
Sphingolipid metabolism								
**Sa**	10.70 ^c^	10.66 ^c^	12.85 ^b^	10.98 ^c^	21.94 ^a^	11.42 ^c^	<0.0001	4.16
**So**	32.70 ^a^	32.88 ^a^	31.42 ^a^	32.36 ^a^	26.11 ^b^	31.74 ^b^	<0.0001	4.4
**Sa/So**	0.33 ^a^	0.32 ^a^	0.41 ^b^	0.34 ^c^	0.84 ^a^	0.36 ^c^	<0.0001	6.53

CON = control; TXF = Toxfin™ Dry; T2 = toxin T-2; FUM = fumonisin. RF = rheumatoid factor (IU/mL); CRP = C-reactive protein (mg/L); HDL-C = high-density lipoprotein cholesterol (mg/dL); Chol = cholesterol (mg/dL); TG = triglycerides (mg/dL); Glu = glucose (mg/dL); UA = uric acid (mg/dL); U = urea (mg/dL); CRE = creatinine (mg/dL); TB = total bilirubin (mg/dL); AST = aspartate aminotransferase (U/L); ALT = alanine aminotransferase (U/L); GGT = gamma-glutamyl transferase (U/L); ALP = alkaline phosphatase (U/L); TP = total protein (g/dL); Alb = albumin (g/dL); CK = creatine kinase (U/L); Ca = calcium (mg/dL); P = phosphorus (mg/dL); SOD = superoxide dismutase (U/mg); CAT = catalase (U/mg); GPx = glutathione peroxidase (U/mg); MDA = malondialdehyde (nmol/mL); Sa = sphinganine (pmol/mL); So = sphingosine (pmol/mL); Sa/So = sphinganine to sphingosine ratio (dimensionless). Different letters within rows indicate significant differences by Tukey’s test at the 5% probability level.

## Data Availability

The data presented in this study are available within the article. Additional data supporting the findings of this study are available from the corresponding author upon reasonable request.
